# Older adults life expectancy in China: Bayesian mortality estimation and spatially heterogeneous determinants

**DOI:** 10.3389/fpubh.2026.1851714

**Published:** 2026-07-06

**Authors:** Ke Hu, Xingjin Yang, Chaojie Li, Xing Zhang, Di Xiao, Mingyang Yu

**Affiliations:** 1 Xiamen Haicang Hospital, Xiamen, Fujian, China; 2 QianDongNanZhou Center for Disease Control and Prevention, QianDongNanZhou, Guizhou, China; 3 Xingtai Center for Disease Control and Prevention, Xingtai, Hebei, China; 4 Nanjing Lishui Dongping Street Health Center, Nanjing, Jiangsu, China; 5 Community Health Service Center of Jiuxian Tongliang District, Chongqing, China; 6 Fuwai Central China Cardiovascular Hospital, Zhengzhou, Henan, China

**Keywords:** Bayesian random effects, older adult life expectancy, geographically weighted regression, multiple linear regression, multiscale geographically weighted regression, spatial error model, spatial heterogeneity, spatial lag model

## Abstract

**Introduction:**

Life expectancy among the older adults is a key indicator of population health, yet provincial disparities in China remain substantial. Estimating older adults mortality is challenged by small sample sizes and spatial dependence, while the determinants of life expectancy may exhibit spatial heterogeneity.

**Methods:**

Using data from the 2015 China National 1% Population Sample Survey, this study first employed Bayesian random effects models to estimate age-specific mortality rates for the older adults aged 60 + across 31 provinces, accounting for spatial correlation and small-sample smoothing. The abridged life table method was then used to calculate provincial life expectancy. Finally, multiple linear regression (MLR), spatial error model (SEM), spatial lag model (SLM), geographically weighted regression (GWR), and multiscale geographically weighted regression (MGWR) were compared to explore the spatially varying effects of socio-environmental factors, including old dependency ratio, healthcare resources, air pollution, education, and pension security.

**Results:**

The Bayesian model incorporating age-region interaction performed best. Older adults life expectancy exhibited a clear east–west gradient, with higher values in eastern and northeastern provinces and lower values in central and western regions. Global regression models showed that old dependency ratio and physician density were positively associated with life expectancy, while PM_10_, education, and pension proportion were not significant. However, GWR suggested potential spatial heterogeneity: old dependency ratio and physician density showed patterns of stronger effects in northern China; PM_10_ exhibited a more negative association in southwestern China; and pension security was significant only in northwestern provinces. GWR outperformed MGWR (*R*^2^ = 0.789 vs. 0.664), likely due to the small sample size, which makes MGWR prone to unstable bandwidth estimation.

**Conclusion:**

Older adults life expectancy in China appears to be characterized by spatial disparities and potentially location-specific determinants. Uniform national policies may be insufficient to address regional health inequalities. Provincial governments might consider tailored strategies for older adults health resource allocation, with northern areas leveraging family and community support, southwestern regions prioritizing air pollution control, and northwestern areas expanding pension coverage.

## 1 Introduction

As healthcare conditions and living standards continue to improve in China, average life expectancy has been rising year by year. However, this increase in life expectancy has been accompanied by an increasingly serious challenge of population aging (1). Given the decline in various bodily functions among the older adults, issues such as disability, cognitive impairment, and chronic diseases have become more prominent, placing a significant burden on families and society in terms of older adults care and medical expenses (2, 3). Scientifically measuring the overall health status of the older adults population across different provinces is crucial for health policy formulation, such as the allocation of regional medical resources (4, 5).

Life expectancy of the older adults refers to the average remaining years of life for a specific age group within the older population, serving as a key indicator for comprehensively evaluating the health status of the older adults in a given province. Estimating and comparing the life expectancy of older adults across different provinces is helpful for promoting the development and improvement of health policies for the older adults. Although life expectancy among the older adults in China has shown a continuous increasing trend, certain disparities exist across provinces. Based on data from the 2010 national census, the province with the highest life expectancy among the older adults was Beijing (22.75 years), while the lowest was Tibet (17.02 years), with a difference of nearly 6 years. Therefore, it is necessary to explore the factors influencing the life expectancy of the older adults in China from a spatial perspective, in order to address the imbalance in life expectancy among older populations across provinces (6).

However, existing research still has two key scientific gaps. First, most studies have focused on describing spatial disparities in life expectancy or simply listing influencing factors, lacking an in-depth exploration of the underlying mechanisms - namely, whether the effects of socio-environmental factors on older adults life expectancy exhibit spatially heterogeneous patterns, and how the direction and intensity of these effects vary across regions. Second, current studies have rarely compared the performance of different spatial regression models (such as global vs. local models, single-scale vs. multi-scale models) in capturing such heterogeneity, making it difficult to determine which analytical framework is more suitable for revealing the complex drivers of older adults life expectancy at the provincial level in China. Clarifying these scientific questions is essential for moving from phenomenological description to mechanistic understanding.

The estimation of life expectancy relies on the measurement of mortality rates. Traditional mortality models generally fall into two categories, namely deterministic and stochastic models, both of which have certain limitations. Deterministic models estimate parameters solely based on historical experience, ignoring the uncertainty of mortality changes over time. Although stochastic models can account for the effects of various random factors, they fail to consider the spatial correlation of mortality rates across provinces. In fact, due to the regional clustering of socioeconomic and environmental factors, mortality levels in adjacent provinces often exhibit spatial autocorrelation (7, 8). Moreover, sample sizes in population health surveys, especially those targeting the older adults, are often small, and directly applying traditional methods to estimate mortality rates may lead to biased results.

The Bayesian random effects model has been increasingly applied in mortality estimation because it can address the correlation of mortality rates across different provinces and age groups, as well as the impact of small sample sizes on mortality estimation (9). This model not only accounts for the correlation of mortality rates between neighboring provinces and age groups, but also smooths the age specific mortality rates for populations with small sample sizes (10), thereby revealing the changing patterns of life expectancy in a more scientific and accurate manner. Therefore, this study employs the Bayesian random effects model to estimate mortality rates for the older adults across various age groups at the provincial level in China, while taking into account the correlation of mortality rates between provinces and age groups. Subsequently, the abridged current life table method is used to estimate the life expectancy of the older adults for each age group in each province.

Currently, there are numerous studies on the factors influencing life expectancy at birth, but relatively few explorations focusing on the older adults. Biological factors and social environmental factors are the main influences on life expectancy. However, because individual biological data are often difficult to obtain, social environmental factors have become the most commonly considered variables (11). Social environmental factors, including economic development, education level, environment, and healthcare availability, have all been shown to have a certain impact on life expectancy (12). For example, economic development can improve life expectancy by influencing living standards and healthcare access (13, 14). Populations with higher education levels tend to have better health awareness and timely access to medical services. Air pollutants such as PM_10_ have a negative effect on life expectancy (15, 16). The dependency ratio also has a negative impact on life expectancy (17).

From the perspective of methods for exploring influencing factors, early studies mostly employed regression analyses that did not account for spatial information, such as multiple linear regression. However, these models failed to utilize spatial information, and their residuals often exhibited spatial autocorrelation (18). To address this issue, researchers subsequently adopted the spatial error model (SEM) and the spatial lag model (SLM) (19–21). The SEM assumes that the error terms of the independent variables in a study region are correlated with those of neighboring regions, whereas the SLM assumes that the dependent variable in neighboring regions exhibits spatial autocorrelation. Although global spatial regression models account for spatial correlation among variables, they overlook the spatial heterogeneity in the effects of various factors on life expectancy across different geographic locations (19).

To this end, Brunsdon and colleagues proposed the geographically weighted regression (GWR) model in 1998, which embeds geographic location into the regression coefficients to reveal how the influence of factors on life expectancy varies by geographic location (22). In recent years, multiscale geographically weighted regression (MGWR), as an extension of GWR, allows different variables to have different bandwidths, further improving the flexibility and accuracy of model fitting (23). Previous studies have employed the GWR model to explore the spatial distribution of the influence intensity of various factors on life expectancy and found that its performance outperforms stepwise regression and global spatial regression models (6, 24).

To address the above scientific gaps, this study systematically investigates two core scientific questions: (1) After accounting for spatial correlation and small-sample smoothing using a Bayesian random effects model, what are the spatial patterns of older adults life expectancy across Chinese provinces? (2) Do the effects of key socio-environmental factors on older adults life expectancy exhibit significant spatial heterogeneity? If so, how do the direction, magnitude, and significance of these effects differ across regions? Furthermore, this study compares the performance of GWR and MGWR in capturing such heterogeneity under a small-sample provincial setting, aiming to identify a more appropriate analytical framework.

In summary, this study first employs the Bayesian random effects model to estimate mortality rates and life expectancy for the older adults across different age groups in each province of China. Subsequently, the GWR model, MGWR model, SEM, and SLM are used to explore the spatial heterogeneity of the effects of social environmental factors on life expectancy among the older adults. The performance of these models in explaining the spatial variation of influencing factors is compared, with the aim of providing a scientific basis for the allocation of healthcare resources and policy formulation in different provinces, and ultimately addressing the imbalance in life expectancy among the older adults across provinces.

## 2 Methods

### 2.1 Data sources

#### 2.1.1 Study scale

This study was conducted at the scale of 31 provincial-level administrative units (provinces, municipalities directly under the central government, and autonomous regions) in mainland China.

#### 2.1.2 Data sources for mortality estimation

The population data and death counts required for mortality estimation of the older adults aged 60 and above in this study were derived from the 2015 National 1% Population Sample Survey, rather than the Seventh National Population Census conducted in 2020. The main reasons are as follows. First, 2020 was the first year of the COVID-19 pandemic, during which the mortality pattern of the Chinese population was significantly impacted by the outbreak, leading to considerable short term fluctuations in mortality data that cannot represent the stable mortality level of the older adults population in normal years. Using the 2020 data could therefore introduce bias into mortality estimates (25). Second, the 2015 National 1% Population Sample Survey is large in scale. However, for certain provinces and the oldest age groups, the sample sizes of age-specific deaths remain relatively small, making mortality estimates susceptible to random fluctuations. The Bayesian random effects model employed in this study is specifically designed to address such small sample data issues, as it can borrow information from neighboring provinces and adjacent age groups to effectively smooth and robustly estimate mortality rates for small samples (26, 27).

#### 2.1.3 Data sources of social environmental factors

Based on previous research and data availability, the following indicators of social environmental factors were selected for an exploratory analysis of factors influencing life expectancy among the older adults: old dependency ratio, number of physicians per 1,000 population, health expenditure per capita, annual average PM_2.5_ concentration, annual average PM_10_ concentration, average years of education among the older adults, and the proportion of older persons whose main source of income is a pension.

Specifically, the old dependency ratio, average years of education among the older adults, and the proportion of older persons whose main source of income is a pension for each province in 2015 were derived from the 2015 China National 1% Population Sample Survey. The number of physicians per 1,000 population and health expenditure per capita by province in 2015 were obtained from the 2016 and 2017 editions of the China Health and Family Planning Statistical Yearbook, respectively. The annual average concentrations of PM_10_ and PM_2.5_ for each province in 2015 were obtained from the environmental bulletins of the respective provinces for the same year (see Table 1).

**Table 1:** Selected variables and their data sources.

Category	Variable	Data sources
Demographic characteristics	Old dependency ratio	China 2015 National 1% Population Sample Survey
Healthcare resources	Number of physicians per 1,000 population	China Health Statistical Yearbook (2016 and 2017 editions for 2015 data)
	Health expenditure per capita	
Environmental quality	PM_2.5_	Provincial Environmental Status Bulletins of 2015
	PM_10_	
Educational attainment	Average years of education among the older adults	China 2015 National 1% Population Sample Survey
Household economic security	Proportion of older persons whose main source of income is a pension	China 2015 National 1% Population Sample Survey

### 2.2 Death underreporting estimation and population adjustment

Although the 2015 China National 1% Population Sample Survey is of high quality, death underreporting still exists. In this study, the death underreporting rate in the sample data was calculated based on the national mortality rate for 2015 published by the National Bureau of Statistics, using the following Equation 1:


$$\text{Death underreporting rate}=\frac{\begin{array}{l} \text{Mortality rate from the}\;2015\;\\ \text{National}\;1\%\text{Population}\;\\ \text{Sample Survey} \end{array}}{\begin{array}{l} \text{National mortality rate}\;\\ \text{published}\;\mathrm{by}\;\text{the National}\;\\ \text{Bureau of Statistics} \end{array}}–1$$ (1)

Since the number of deaths serves as the dependent variable in the Bayesian random effects model and follows a binomial distribution, directly adjusting the death counts may alter their probability distribution. Therefore, this study kept the death counts unchanged and only adjusted the total population accordingly. The adjustment was applied uniformly at the national level, as reliable province-specific underreporting estimates were not available. Specifically, the survey population for each province was adjusted using the national underreporting rate from Equation 1, providing a corrected population denominator for subsequent mortality estimation and life expectancy calculations.

### 2.3 Mortality estimation using the Bayesian random effects model

#### 2.3.1 Model specification

The Bayesian random effects model was used to estimate mortality rates for different age groups across provinces in China. Provinces are denoted by *i*, where *i* = 1 to 31, and *x* represents the age group. Given that the number of deaths at age 90 and above in the sample data is relatively small, leading to unstable mortality estimates, the population aged 60 and above was divided into 7 age groups, where *x* = 1, 2, …, 7. In Equation 2, *m* denotes the mortality rate estimated by the Bayesian model (i.e., the probability of death within a specific age group of the older adults population, reflecting the level of mortality risk in that group), *p* represents the adjusted total population, and *y* represents the number of deaths.

Death is not a rare event among the older adults. Therefore, this study assumes that the number of deaths(*y*) follows a binomial distribution (28), as specified in Equation 2:


$$y_{\mathrm{ix}}\sim \mathrm{Bin}(m_{\mathrm{ix}},p_{\mathrm{ix}})$$ (2)

Considering the effects of regional factors and age group factors on mortality, we constructed the following random effects model (Model 1), as specified in Equation 3:


$$\text{Model}\;1:\text{logit}(m_{\mathrm{ix}})=s_{i}+h_{x}+\alpha$$ (3)

In Equation 3, *s_i_* represents the regional effect, capturing the correlation of mortality rates across regions, *h_x_* represents the age group effect, capturing the correlation of mortality rates across age groups, *α* is the intercept term.

Since the age effect and regional effect alone may not fully account for the extra binomial variation, this study introduced an age by province interaction effect (z_ix_) based on Model 1, resulting in Model 2 as specified in Equation 4:


$$\text{Model}\;2:\text{logit}(m_{\mathrm{ix}})=s_{i}+h_{x}+z_{\mathrm{ix}}+\alpha$$ (4)

#### 2.3.2 Prior specification of parameters

Based on previous experience in Bayesian mortality studies, the prior distributions of the parameters in Model 1 and Model 2 were specified as shown in Table 2.

**Table 2:** Prior distributions of parameters.

Parameter	Prior distribution
*h*_x_	*h*_x_ *~ N(h*_x-1_, *W*_1_*)*
*T*_1_	*T*_1_ *~* Gamma (1, 0.001)
*s_i_*	$s_{i}|s_{\left[-i\right]}\sim N(\frac{1}{\mathrm{nu}m_{i}}\sum_{j\in \mathrm{ad}j_{i}}s_{j},\frac{W_{2}}{\mathrm{nu}m_{i}})$
*T*_2_	*T*_2_ *~* Gamma (0.5,0.0005)
*z*_ix_	*z*_ix_ *~ N(0*, *U*_x_*)*
*C*_x_	*C*_x_ *~* Gamma (1, 0.001)
*α*	*α ~ N(−5*,*100)*

*T_1_ = 1/ W_1_; T_2_ = 1/ W_2_; C_x_ = 1/U_x_*.

Given the correlation of mortality rates across age groups, the age group effect *h_x_* follows a first-order normal autoregressive random walk model with variance *W*_1_ (29), i.e., *h_x_* ~ *N*(*h*_*x-*1_, W_1_), and its precision parameter *T*_1_ (the reciprocal of the variance, i.e., *T*_1_ = 1/ *W*_1_) follows a Gamma distribution,i.e., T_1_ ~ Gamma (1, 0.001) (29).

The prior distribution of the regional effect *s*_i_ was specified as a normal conditional autoregressive model (28), as detailed in Equation 5:


$$s_{i}|s_{\left[-i\right]}\sim N(\frac{1}{\sum_{j}w_{\mathrm{ij}}}\sum_{i\neq j}w_{\mathrm{ij}}s_{j},\frac{W_{2}}{\sum_{j}w_{\mathrm{ij}}})$$ (5)

Where *W*_2_ is the variance of the regional effect *s*_i,_ and its precision parameter *T*_2_ (the reciprocal of the variance, i.e., T_2_ = 1/ *W*_2_) follows a Gamma distribution, i.e. T*_2_* ~ Gamma (0.5, 0.0005). The term *w*_ij_ represents the spatial weight, with *w_ij_* = 1, if two provinces are adjacent, and *w_ij_* = 0 otherwise.

Under the adjacency-based spatial weight matrix required by the car.normal distribution in OpenBUGS software, Hainan Province has no neighboring provinces. Consequently, its regional random effect was assigned an unconditional prior and estimated solely from its own data, without incorporating spatial information from other provinces.

Let *num*_i_ be the number of neighboring provinces of province *i*, and let *adj*_i_ be the set of those neighboring provinces. The prior distribution of *s*_i_ can be further expressed as Equation 6.


$$s_{i}|s_{\left[-i\right]}\sim N(\frac{1}{\mathrm{nu}m_{i}}\sum_{j\in \mathrm{ad}j_{i}}s_{j},\frac{W_{2}}{\mathrm{nu}m_{i}})$$ (6)

The age region interaction effect *Z_ix_* in Model 2 follows a normal distribution with a mean of 0 and variance *U*_x_ (29, 30). The reciprocal of the variance *U_x_* denoted as *C_x_*,follows a Gamma distribution, i.e., C*_x_ ~* Gamma (1, 0.001) (29). The intercept term *α* follows a normal distribution with a mean of −5 and a variance of 100 (31).

#### 2.3.3 Model convergence diagnosis and model evaluation

The above models were estimated using Bayesian inference via the Markov Chain Monte Carlo (MCMC) method. Two independent chains were set. Each chain first underwent 10,000 pre iterations, followed by another 50,000 iterations after burn- in, and finally the posterior mean and 95% credible interval of each parameter were obtained.

Model convergence was diagnosed using the variance ratio method (Brooks-Gelman-Rubin, 1998) (32) combined with autocorrelation plots. The variance ratio method, also known as the Brooks-Gelman-Rubin diagnostic, is suitable for testing the convergence of two Markov chains. This statistic is an improvement by Brooks and Gelman (1998) on the method proposed by Gelman and Rubin (1992) (33). The calculation formula for the diagnostic indicator *R* is shown in Equation 7.


R=V/WSS. (7)

In Equation 7, *V* represents the pooled posterior variance, and *WSS* represents the within sample mean variance. If the diagnostic indicator *R* for a given parameter fluctuates around 1, it indicates that the model has converged.

Brooks and Gelman (1998) (32) proposed a more convenient convergence diagnostic method based on this approach. The principle is as follows: the length of the 100(1-*α*)% interval is calculated for each Markov chain. For m chains, *m* interval lengths are obtained, and their average is denoted as l. Next, the length of the 100(1-*α*)% interval is calculated using the simulated values from all m chains, denoted as *L*. The diagnostic indicator *R_c_* can then be calculated using Equation 8.


$$R_{c}=L/l.$$ (8)

The diagnostic criterion is the same as that of the Gelman and Rubin method. If the diagnostic indicator *R_c_* for a given parameter fluctuates around 1, the model can be considered to have converged.

In addition, if the autocorrelation coefficient in the autocorrelation plot for a given parameter approaches 0, this also indicates model convergence (32). Given the large number of parameters in the model, it is sufficient to examine the convergence of only the main parameters to determine whether the model has converged.

After model convergence, Model 1 and Model 2 need to be evaluated. We used the Deviance Information Criterion (*DIC*) and the effective number of parameters *pD* for judgment. The model with smaller values of both *DIC* and *pD* is considered the optimal model. The relationship between *DIC* and *pD* is shown in Equation 9:


DIC=Dbar+pD. (9)

Where *Dbar* is the posterior mean of the Bayesian deviance, and *pD* is the effective number of parameters. If the difference in *DIC* values between two models is greater than 7, the model with the smaller *DIC* is significantly better than the other. If the difference is between 3 and 7, the performance difference between the two models is not obvious. If the difference is less than 3, the two models perform similarly, and in this case, the model with the smaller *pD* should be preferred to reduce model complexity and improve computational efficiency.

#### 2.3.4 Sensitivity analysis

For the precision parameters of the age group effect, regional effect, and age region interaction term, this study referred to the study by Congdon (29), and considered the following prior specification: *T*_1_ ~ Gamma (1, 0.0001), *T*_2_ ~ Gamma (1, 0.0001) and *α* ~ flat()(the corresponding model is named Model 3).

These were compared with the original prior specification: *T*_1_ ~ Gamma (1, 0.001), *T*_2_ ~ Gamma (0.5, 0.0005) and *α* ~ *N*(−5,100). If the posterior means of the parameters in the optimal model (between Model 1 and Model 2) and Model 3 are similar, the model can be considered insensitive to the prior specification and thus robust.

### 2.4 Estimation of life expectancy among the older adults using the life table method

After obtaining the posterior mean of age-specific mortality rates for each province, we used the abridged life table method to estimate life expectancy at age 60 (the average remaining years of life for the older adults, E_60_) in each province. The life table method is a statistical table constructed based on the age specific mortality rates of a specific population. Because this method accounts for mortality conditions across different age groups, it can be used to compare populations across different provinces and time periods.

### 2.5 Analysis of the heterogeneity of social environmental factors influencing life expectancy among the older adults

All continuous variables were standardized to have a mean of zero and a standard deviation of one prior to model fitting. This ensures that the resulting regression coefficients are comparable across variables with different units of measurement.

#### 2.5.1 Multiple linear regression (MLR)

This study first used multiple linear regression as a baseline model to analyze the combined effects of multiple independent variables on a single dependent variable. The basic form of the model is shown in Equation 10:


$$Y=\beta_{0}+\beta_{1}X_{1}+\beta_{2}X_{2}+\ldots +\beta_{p}X_{p}+\varepsilon$$ (10)

In this model, *Y* represents the life expectancy of the older adults (dependent variable, calculated from life table method in Section 2.4), *X*_1_ to *X*_p_ denote the social environmental factors, *β*_0_ is the intercept term, *β*_1_ to *β*_p_ are the regression coefficients, and *ε* is the random error term, which follows a normal distribution with a mean of zero. The explanatory power of the model was assessed using the coefficient of determination (*R*^2^). The trade off between model complexity and goodness of fit was evaluated using the Akaike Information Criterion (AIC).

Multicollinearity was tested using the variance inflation factor (VIF), with a VIF value exceeding 10 indicating significant multicollinearity that may compromise the reliability of the model (34).

#### 2.5.2 Global spatial regression models

The global spatial regression models employed a first-order Queen contiguity spatial weight matrix, which defines neighboring relationships based on shared boundaries or vertices.

##### 2.5.2.1 Spatial error model (SEM)

The spatial error model is used to account for spatial autocorrelation present in the regression residuals (21). Its basic formulation is given in Equations 11, 12:


$$Y=X_{\beta }+u$$ (11)

$$u=\lambda W_{u}+\varepsilon$$ (12)

In this model, *λ* represents the spatial error coefficient, and *W*_u_ denotes the spatially lagged error term. By explicitly incorporating the spatial dependence structure into the error term, this model effectively addresses the estimation bias arising from spatial autocorrelation in traditional regression analysis.

##### 2.5.2.2 Spatial lag model (SLM)

The spatial lag model captures spatial dependence effects by incorporating a spatially lagged term of the dependent variable, effectively characterizing the interaction mechanisms between neighboring spatial units (35). The specific form of this model is shown below in Equation 13:


$$Y=\rho W_{Y}+X_{\beta }+\varepsilon$$ (13)

In this model, *ρ* represents the spatial autoregressive coefficient, which quantifies the strength of spatial dependence. *W* denotes the row standardized spatial weight matrix defining the adjacency relationships; and *W*_Y_ constitutes the spatially lagged dependent variable. The SLM framework explicitly accounts for spatial spillover effects, meaning that the value of the life expectancy of the older adults at a given location is influenced by the values of that variable in neighboring locations, while the vector of coefficients *β* controls for the effects of local explanatory variables.

#### 2.5.3 Local spatial regression models

##### 2.5.3.1 Geographically weighted regression (GWR)

The geographically weighted regression model addresses spatial non-stationarity through local regression analysis. Unlike traditional global regression, it allows regression coefficients to vary across spatial locations (36). The basic formula of the GWR model is shown below in Equation 14:


$$Y_{i}=\beta_{0}(u_{i},v_{i})+\sum_{k=1}^{m}\beta_{k}(u_{i},v_{i})x_{\mathrm{ik}}+\varepsilon_{i}$$ (14)

In this model, (*u*ᵢ, *v*ᵢ) represent the spatial coordinates of observation *i*, and *β*_k_(uᵢ, vᵢ) denotes the regression coefficient that varies by location. The model computes spatial weights using a distance decay function and estimates the parameters through weighted least squares. In this study, GWR employed a fixed Gaussian kernel, consistent with the specification used in MGWR, to ensure methodological comparability.

##### 2.5.3.2 Multiscale geographically weighted regression (MGWR)

MGWR extends GWR by allowing different explanatory variables to operate at different spatial scales (37, 38). Its formula is shown below in Equation 15:


$$Y_{i}=\beta_{0}\;(u_{i},v_{i};bw_{0}\;)+\sum_{k=1}^{m}\beta_{k}\;(u_{i},v_{i};bw_{k}\;)x_{\mathrm{ik}}+\epsilon_{i}\;$$ (15)

In this model, *b*_wk_ represents the bandwidth parameter for the *k*_th_ variable. MGWR determines the optimal bandwidth for each variable through an iterative back fitting algorithm, thereby enabling a more accurate characterization of complex spatial dependence patterns. Compared with GWR, MGWR offers superior performance by eliminating the estimation bias caused by a single bandwidth, identifying variable specific spatial scales, and improving the accuracy of interpretation.

In this study, a fixed Gaussian kernel function was adopted, and bandwidth selection was performed based on the cross-validation (CV) criterion. Given that provincial administrative units are relatively evenly distributed in terms of geographic extent and density across the study area, the fixed Gaussian kernel is methodologically appropriate because it assumes a constant spatial influence scale throughout the study region. Under this condition, the choice of kernel function generally has little impact on coefficient estimation, and the fixed kernel has been widely adopted in similar provincial level health geography studies.

### 2.6 Software

The spatial weight matrix for each province was generated by importing the national map into OpenBUGS 3.2.3 using QGIS 3.10. The Bayesian random effects models (Model 1, Model 2, and Model 3) were implemented using OpenBUGS 3.2.3. MLR, SEM, and SLM were performed using GeoDa 1.22. GWR and MGWR were implemented using MGWR 2.2. All spatial mapping was conducted using ArcGIS 10.2. The base map was sourced from the National Platform for Common Geospatial Information Services (approval number: GS(2024)0650). A two-tailed *p* < 0.05 was considered statistically significant.

## 3 Results

### 3.1 Mortality estimation

#### 3.1.1 Convergence assessment

**Figure 1:**
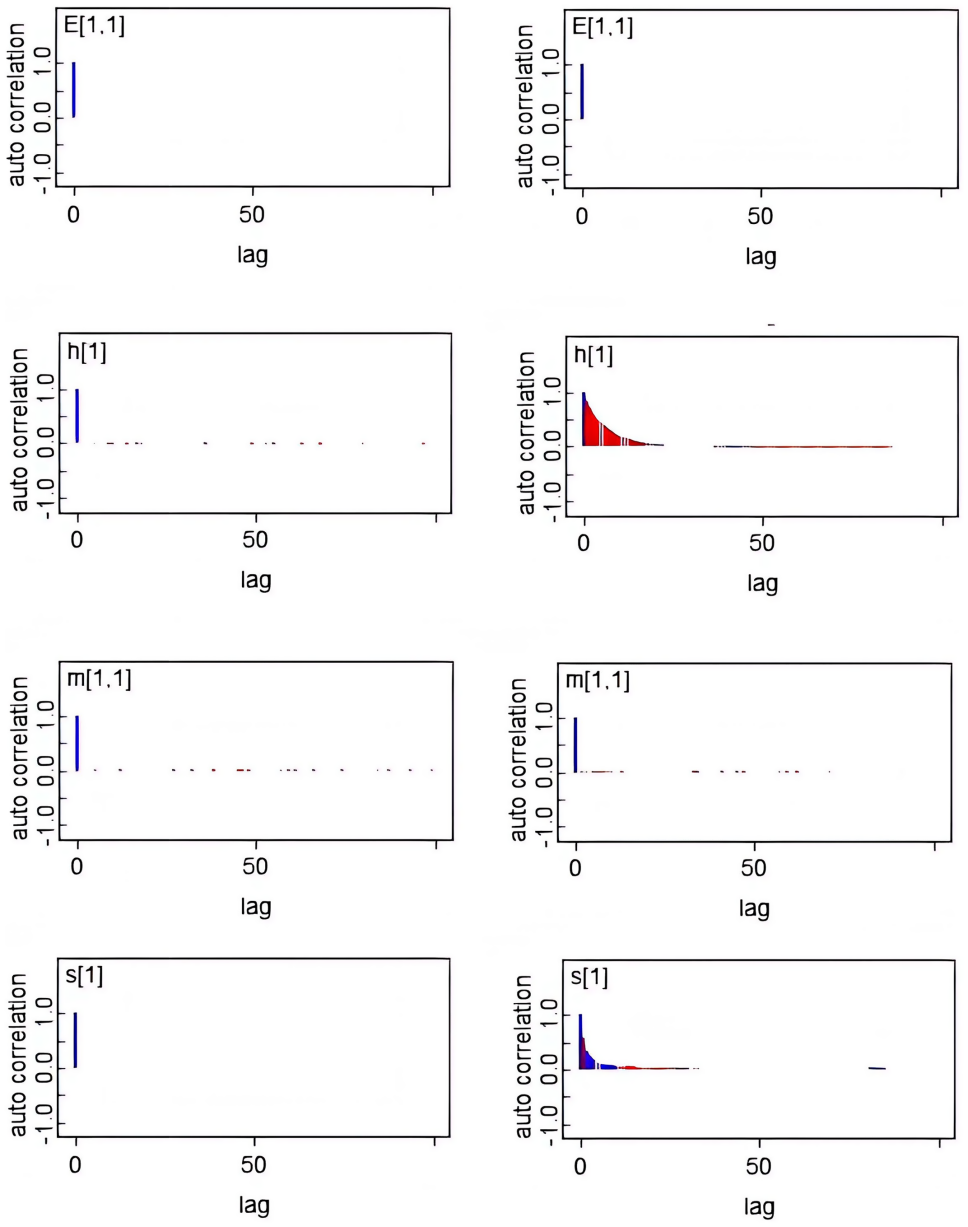
Autocorrelation plots of key parameters for Model 1 (left) and Model 2 (right).

**Figure 2:**
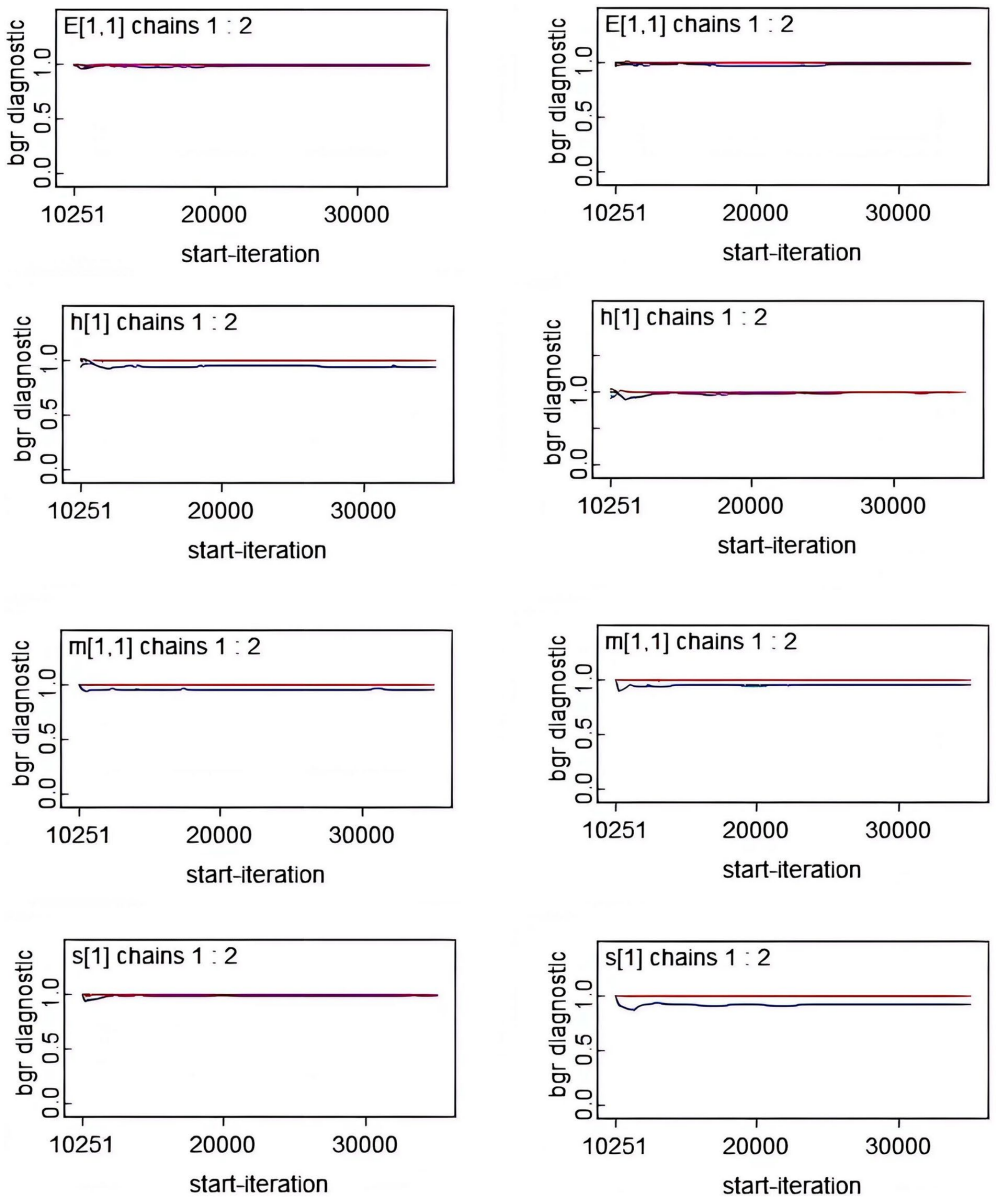
Brooks-Gelman-Rubin diagnostic plots of key parameters for Model 1 (left) and Model 2 (right).

Before calculating the posterior means and 95% credible intervals of the parameters, the convergence of Model 1 and Model 2 needed to be assessed. After 10,000 pre-iterations and another 50,000 iterations after burn-in for both models, the autocorrelation coefficients of the key parameters (*E*, *h*, *m*, *s*) approached zero (Figure 1), indicating that the models had essentially converged. The Brooks-Gelman-Rubin diagnostic plots (Figure 2) showed that the diagnostic indicator *R*_c_ (red line) fluctuated around 1, further confirming good convergence of both models, allowing for a performance comparison to select the optimal model.

#### 3.1.2 Model performance evaluation

Table 3 presents the performance diagnostic criteria for Model 1 and Model 2. The DIC value of Model 2 was substantially lower than that of Model 1, indicating significantly superior performance. Although the effective number of parameters (*pD*) of Model 1 was smaller than that of Model 2, the *pD* of Model 1 was negative, suggesting a certain degree of conflict between its model assumptions and the data, which may be attributed to unaccounted errors in the fitting process. In summary, this study selected Model 2 (which incorporates the age-region interaction effect) as the mortality model for estimating the posterior means of mortality rates and for analyzing the effects of age groups and regional factors on mortality.

**Table 3:** Model performance diagnostic criteria for Model 1 and Model 2.

Diagnostic criterion	Model 1	Model 2
*DIC*	2372.00	1886.00
*pD*	−2.91	74.37

#### 3.1.3 Age group effect of mortality (hx)

**Figure 3:**
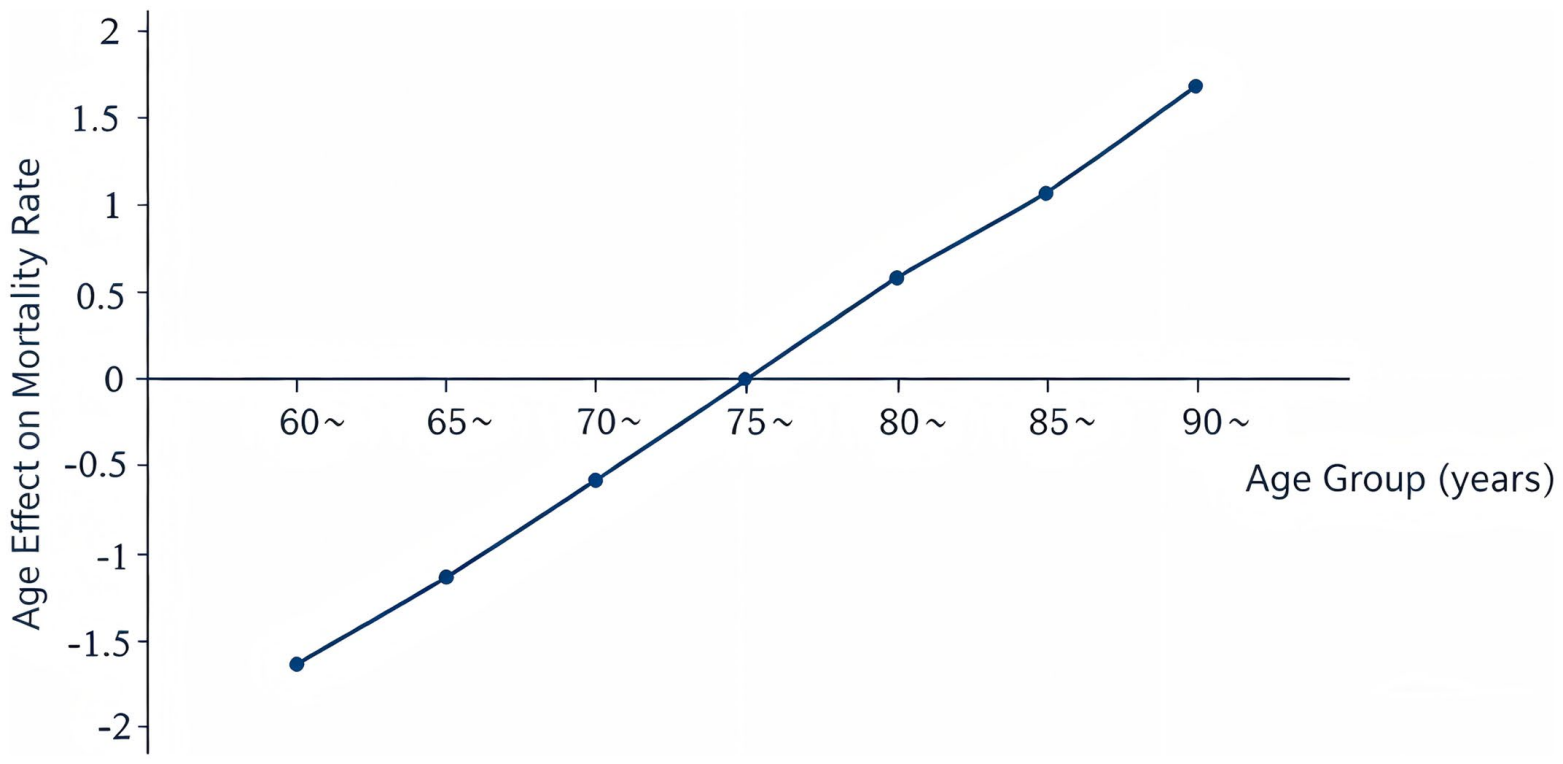
Effect of age on mortality.

Mortality rates change with increasing age, and this pattern is captured by the parameter *h*_x_ in the model. The results are shown in Figure 3.

Figure 3 presents the posterior means of *h*_x_ for each age group. As shown in the figure, the youngest older adults (aged 60–65 years) had the lowest mortality risk. As age increased, the posterior mean of *h*_x_ gradually increased, indicating a clear upward trend in mortality risk, with those aged 90 years and above facing the highest mortality risk.

#### 3.1.4 Regional effect of mortality (si)

**Figure 4:**
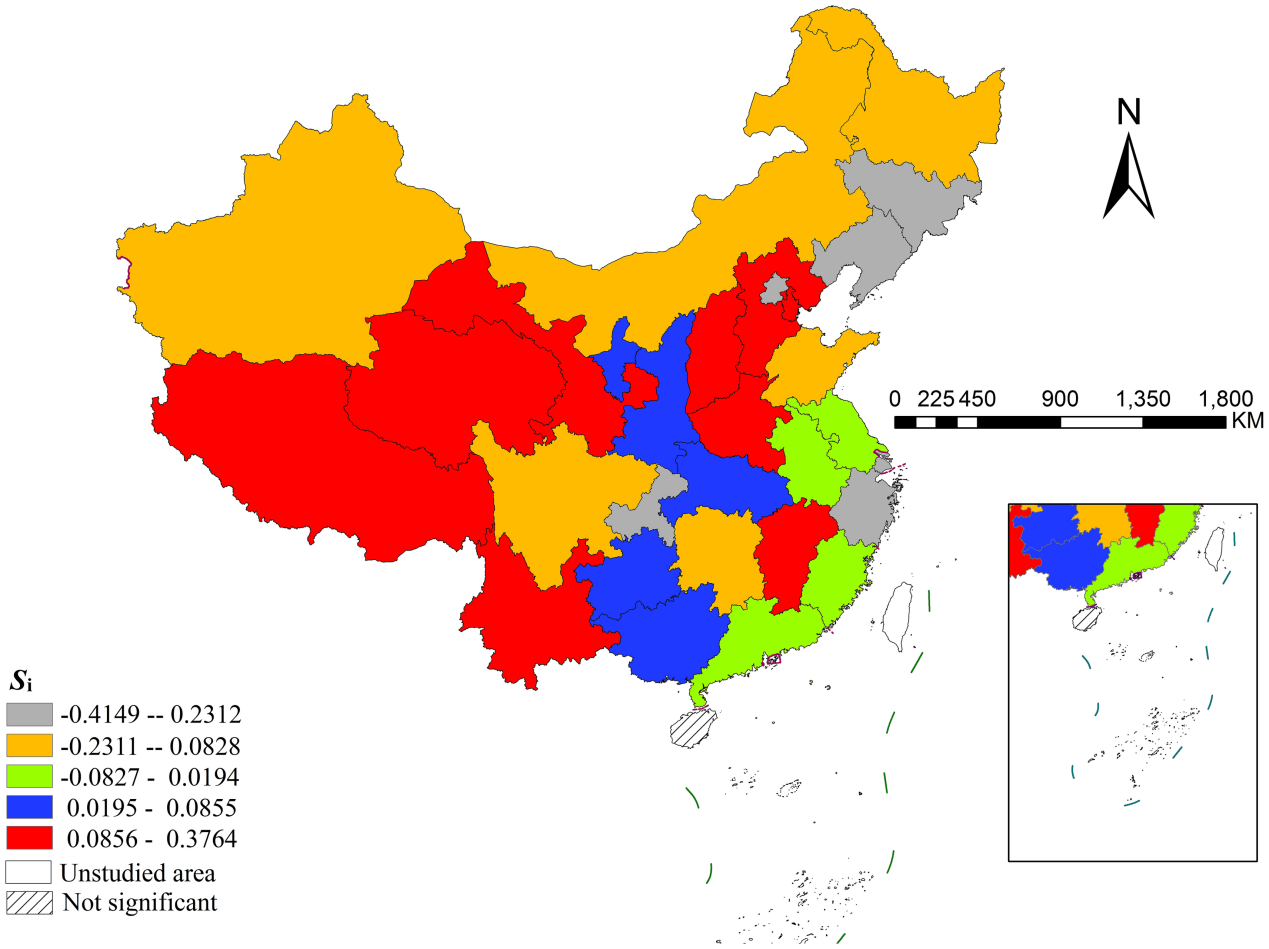
Regional effect on mortality.

Mortality risk in each province is influenced by two factors: first, its own social and environmental conditions, and second, the mortality risk of neighboring provinces (due to similar social and environmental conditions). This influence is termed the “regional effect” and is represented by the parameter *s*_i_, where *s*_i_ > 0 indicates high mortality risk and *s*_i_ < 0 indicates low mortality risk (see Figure 4).

Figure 4 shows that the regional effect exhibits spatial clustering. In developed eastern provinces (e.g., Shanghai and Zhejiang), *s*_i_ was negative, indicating low mortality risk. The three northeastern provinces also had lower *s*_i_ values than the central and western regions, with relatively low mortality risk. In contrast, underdeveloped central and western provinces (e.g., Henan, Shanxi, and Tibet) had positive *s*_i_ values, indicating higher mortality risk, reflecting that less developed areas experience mutual influence of high mortality rates due to poor local conditions and spatial clustering effects.

#### 3.1.5 Sensitivity analysis

**Figure 5:**
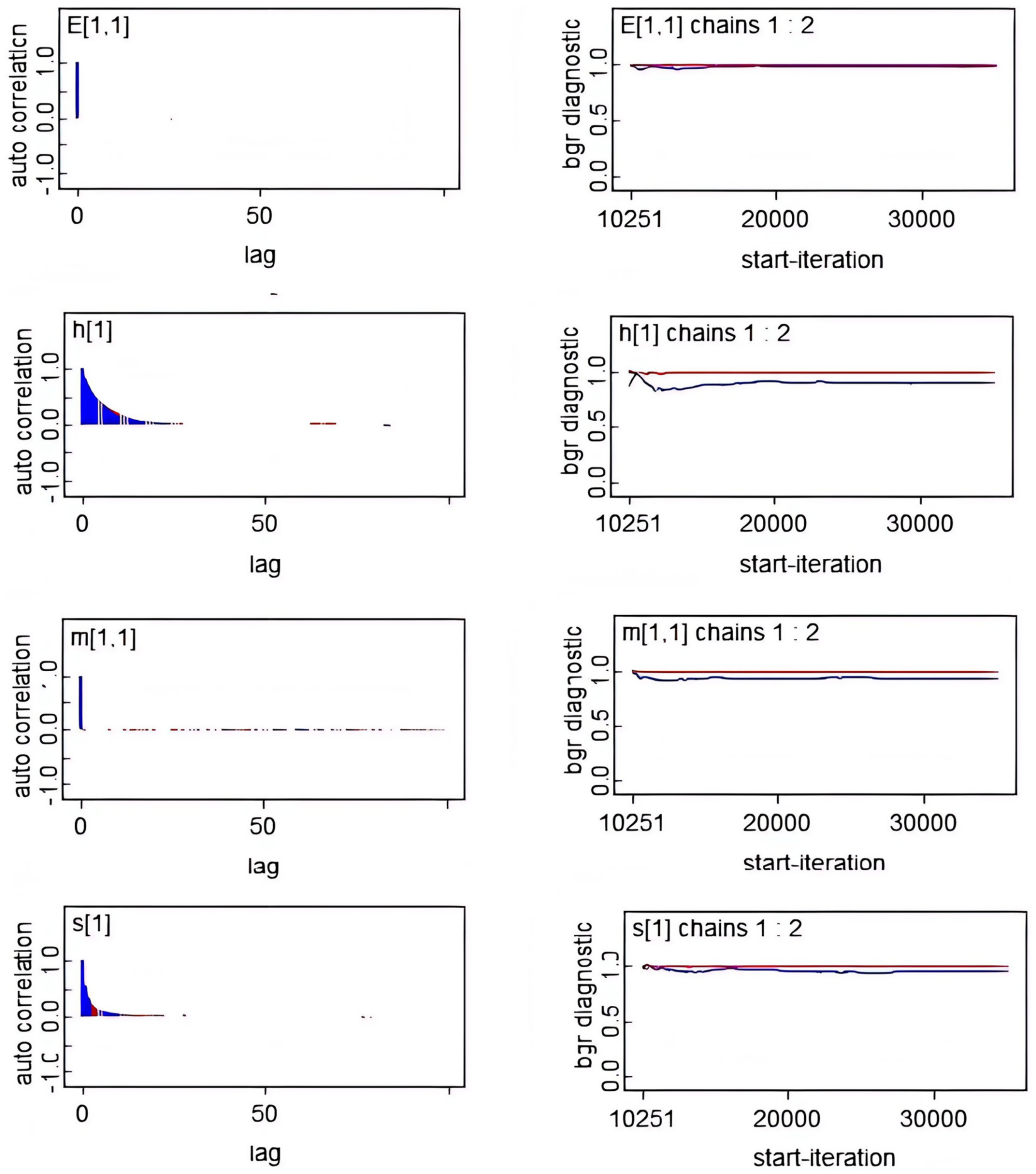
Autocorrelation plot (left) and Brooks-Gelman-Rubin diagnostic plot (right) of key parameters for Model 3.

After 10,000 pre-iterations and another 50,000 iterations after burn-in for Model 3, the autocorrelation coefficients of the key parameters (*E*, *h*, *m*, *s*) approached zero, and the Brooks-Gelman-Rubin diagnostic indicator *R*_c_ fluctuated around 1 (Figure 5), indicating that the model had converged. Table 4 shows that the differences in *DIC* and *pD* between Model 2 and Model 3 were both within 7, indicating no substantial performance difference between the two models. The posterior means of all parameters were nearly identical, suggesting that the model was relatively robust to prior specifications. Model 2 had lower *DIC* and *pD* values than Model 3, indicating a slightly better fit. Considering both goodness-of-fit and model complexity, the original prior specification (Model 2) was deemed more appropriate.

**Table 4:** Comparison of key parameters and goodness-of-fit between Model 2 and Model 3.

Parameter	Model 2	Model 3
*E*_1,1_	22.63000	22.63000
*h*_1_	−1.62700	−1.62700
*s*_1_	−0.17880	−0.17880
*m*_1,1_	0.01026	0.01026
*DIC*	1886.00000	1889.00000
*pD*	74.37000	76.73000

### 3.2 Life expectancy of the older adults and its spatial distribution

**Figure 6:**
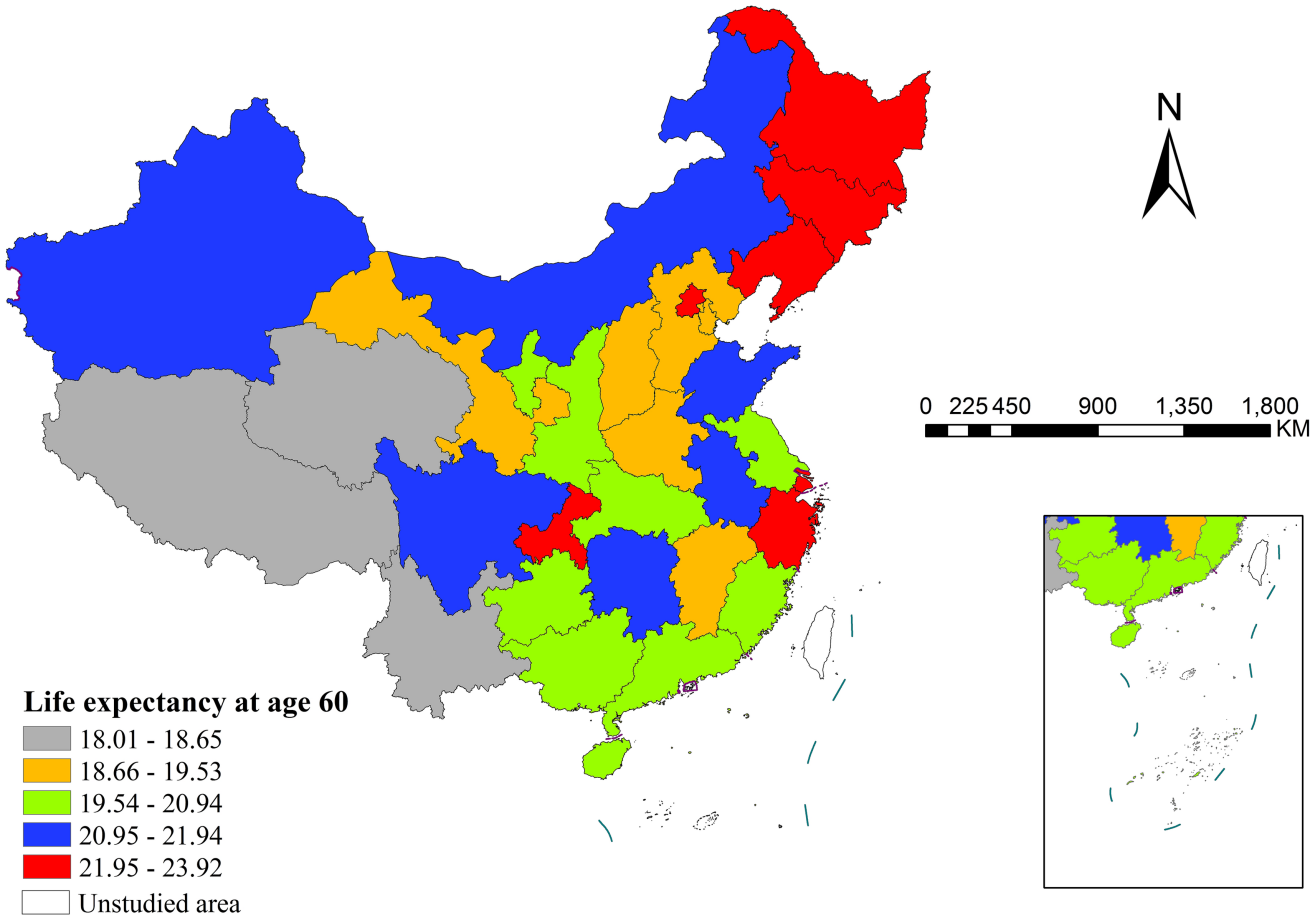
Spatial distribution of life expectancy for the population aged 60 years in China (2015).

Figure 6 presents the spatial distribution of life expectancy for the population aged 60 years across provinces in China, revealing significant regional disparities. The highest levels were observed in Northeast China, the Beijing-Tianjin region, and the eastern coastal areas, while the lowest levels were found in parts of the Northwest and Southwest. Overall, life expectancy exhibited a decreasing gradient from east to west and from coastal to inland regions, reflecting the combined effects of economic development, healthcare resources, and geographical environment on the health status of the older adults population.

### 3.3 Heterogeneity of social environmental factors influencing life expectancy among the older adults

#### 3.3.1 Variable collinearity test

This study used the VIF to test for global multicollinearity among the seven variables (Table 5). The results showed that the VIF of PM_2.5_ exceeded 10, indicating severe collinearity, so it was excluded. The VIF of health expenditure per capita approached 10. To avoid affecting the fitting of the GWR model, it was also excluded. After removing these two variables, the VIF of the remaining variables were all below 4 (Table 6), indicating no severe global multicollinearity.

**Table 5:** Collinearity diagnostics of the selected variables.

Variable	VIF
Old dependency ratio	2.63
Number of physicians per 1,000 population	5.45
Health expenditure per capita	9.95
PM_2.5_	14.74
PM_10_	9.15
Average years of education among the older adults	4.99
Proportion of older persons whose main source of income is a pension	8.25

**Table 6:** Collinearity diagnostics of the selected variables after removing PM_2.5_ and health expenditure per capita.

Variable	VIF
Old dependency ratio	1.30
Number of physicians per 1,000 population	2.74
PM_10_	1.20
Average years of education among the older adults	3.35
Proportion of older persons whose main source of income is a pension	3.85

#### 3.3.2 Global regression model results

Table 7 presents the estimation results of the spatial error model (SEM), spatial lag model (SLM), and multiple linear regression (MLR). The results showed that the coefficient of the old dependency ratio was positive and significant (*p* < 0.001) across all three models, indicating a stable positive association with life expectancy. The number of physicians per 1,000 population was also significantly positive in both SEM and SLM. PM_10_, average years of education among the older adults, and the proportion of older persons whose main source of income is a pension were not significant in any of the models. Furthermore, the spatial dependence parameters were not significant in either SEM (*λ* = 0.0605, *p* = 0.8138) or SLM (*ρ* = 0.0158 *p* = 0.9375), suggesting that global spatial autocorrelation was relatively weak.

**Table 7:** Results of the SEM, SLM and MLR.

Variable	SEM			SLM			MLR		
	Coefficient	*p*-value	Std. error	Coefficient	*p*-value	Std. error	Coefficient	*p*-value	Std. error
Intercept	−0.0020	0.9862	0.1155	−0.0007	0.9952	0.1090	−0.0000	1.0000	0.1212
Old dependency ratio	0.4567	0.0003	0.1258	0.4589	0.0003	0.1263	0.4595	0.0003	0.1404
Number of physicians per 1,000 population	0.4090	0.0269	0.1848	0.3991	0.0298	0.1837	0.3963	0.0635	0.2041
PM_10_	−0.2117	0.0777	0.1200	−0.2070	0.0880	0.1213	−0.2070	0.1372	0.1348
Average years of education among the older adults	0.0327	0.8715	0.2019	0.0273	0.8930	0.2028	0.0259	0.9056	0.2255
Proportion of older persons whose main source of income is a pension	0.2104	0.3295	0.2158	0.2200	0.3112	0.2172	0.2217	0.3678	0.2416
Spatial dependence parameters (ρ in SLM, λ in SEM)	λ = 0.0605	0.8138	/	ρ = 0.0158	0.9375	/	/	/	/

In terms of model goodness-of-fit (Table 8), the coefficients of determination (*R*^2^) for SLM, SEM, and MLR were 0.6206, 0.6214, and 0.6206, respectively, which were very close to each other. The AIC values were 70.91, 68.87, and 68.92, respectively, with SEM and MLR performing slightly better than SLM. Overall, the three global models exhibited similar fitting performance.

**Table 8:** Goodness-of-fit of the five models.

Goodness-of-fit	MGWR	GWR	SLM	SEM	MLR
*R*^2^	0.6640	0.7890	0.6206	0.6214	0.6206
AIC	69.271	62.307	70.9123	68.8693	68.9165
Log likelihood	−27.059	−19.904	−28.4561	−28.4346	−28.4582

Although the spatial dependence parameters in SEM and SLM were not statistically significant, this does not necessarily imply the absence of spatial effects in the data. Global spatial models assume spatial stationarity, which may obscure locally varying relationships. Therefore, we further employed local spatial regression models, i.e., GWR and MGWR, to capture potential spatial heterogeneity that global models failed to detect.

#### 3.3.3 Local spatial regression model results

**Figure 7:**
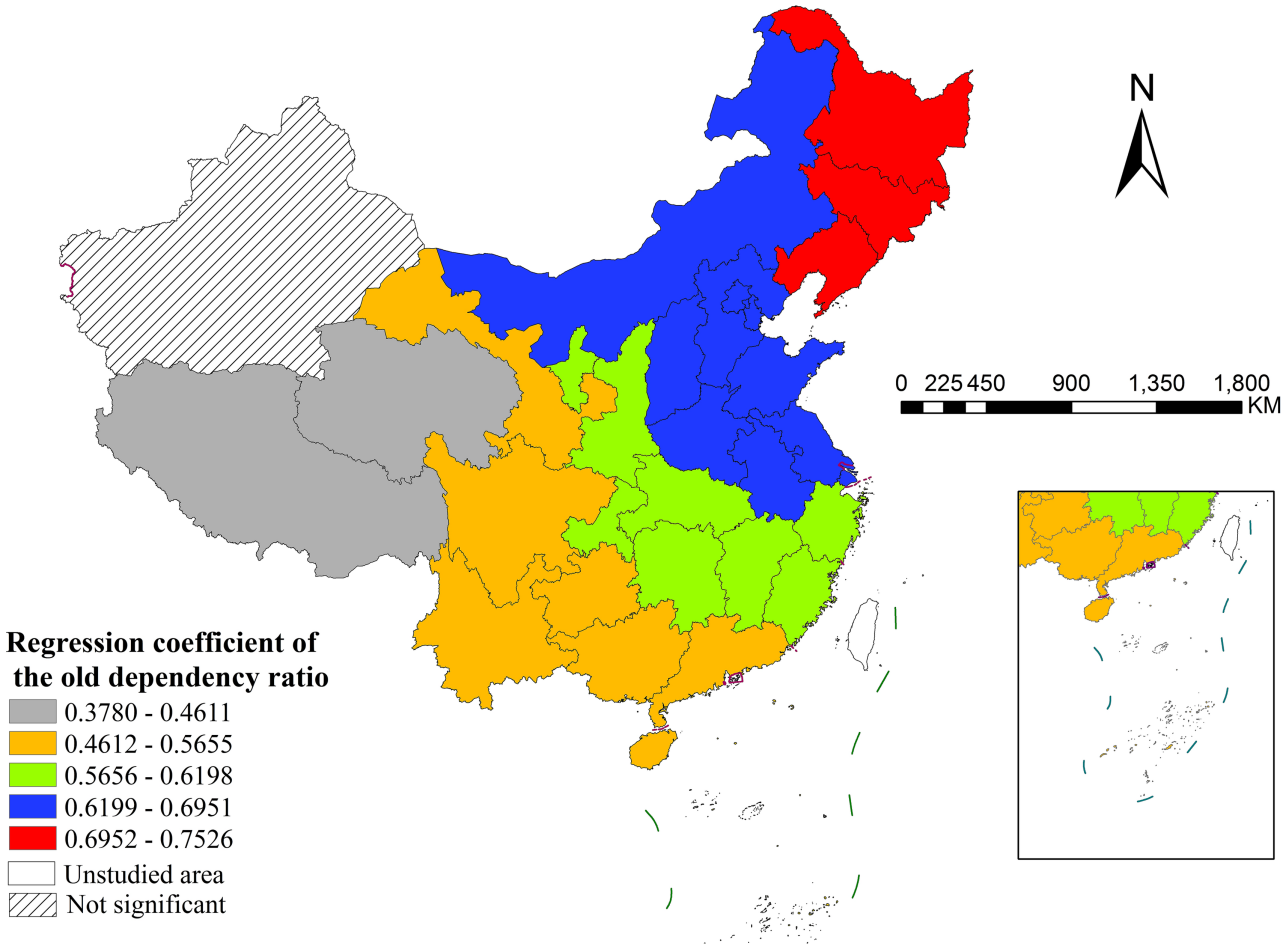
Spatial distribution of regression coefficients for old dependency ratio.

**Figure 8:**
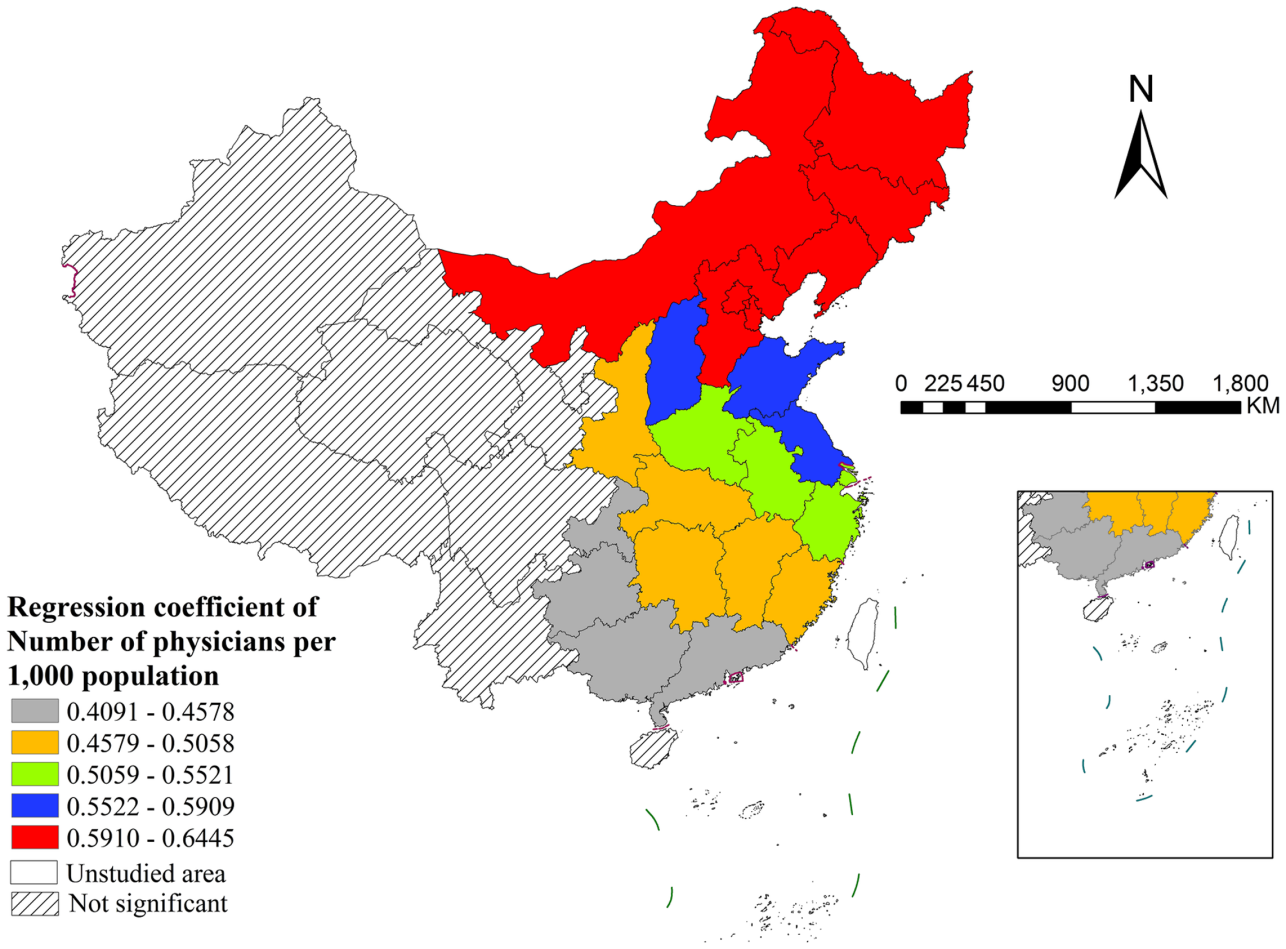
Spatial distribution of regression coefficients for number of physicians per 1,000 population.

**Figure 9:**
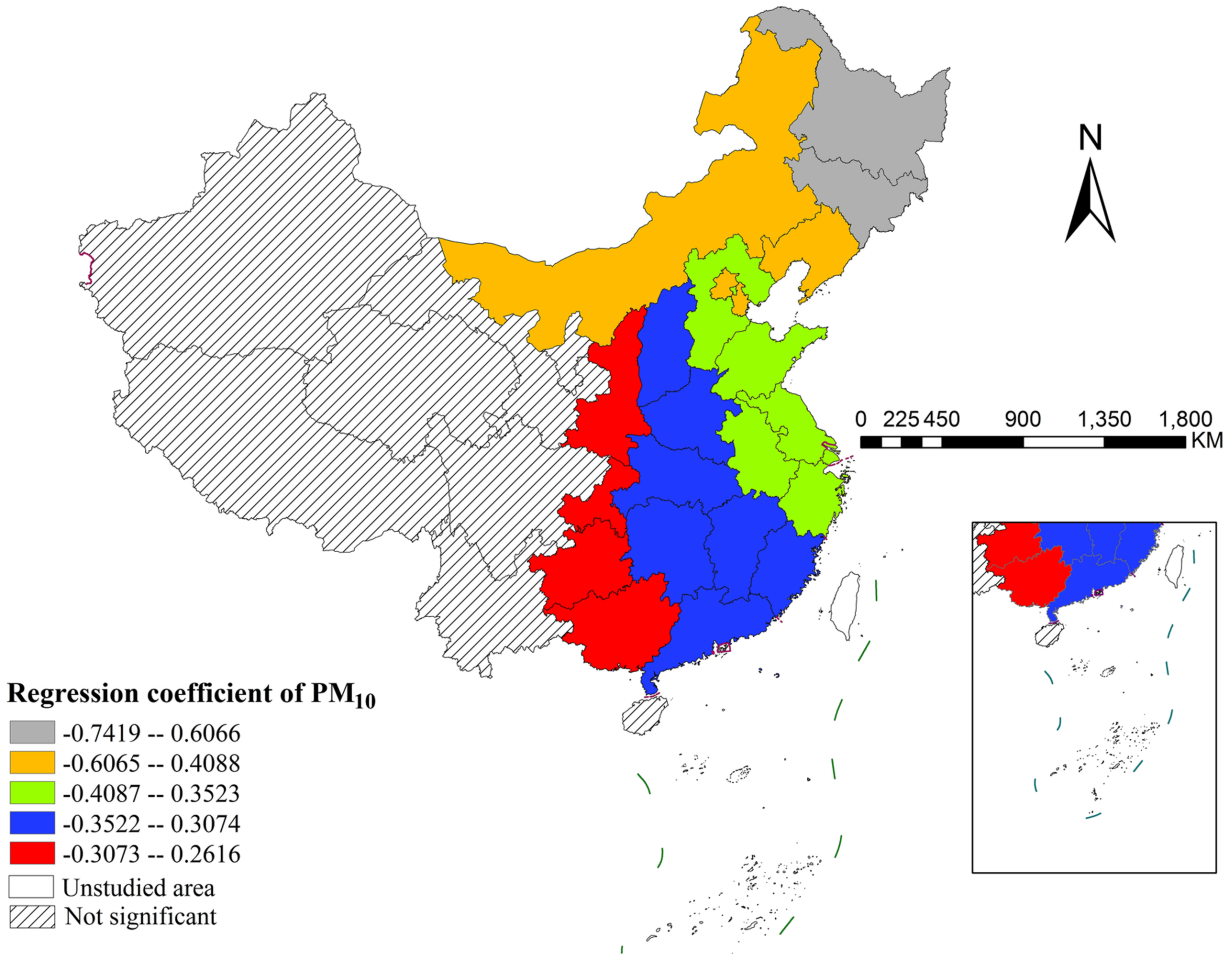
Spatial distribution of regression coefficients for PM10.

**Figure 10:**
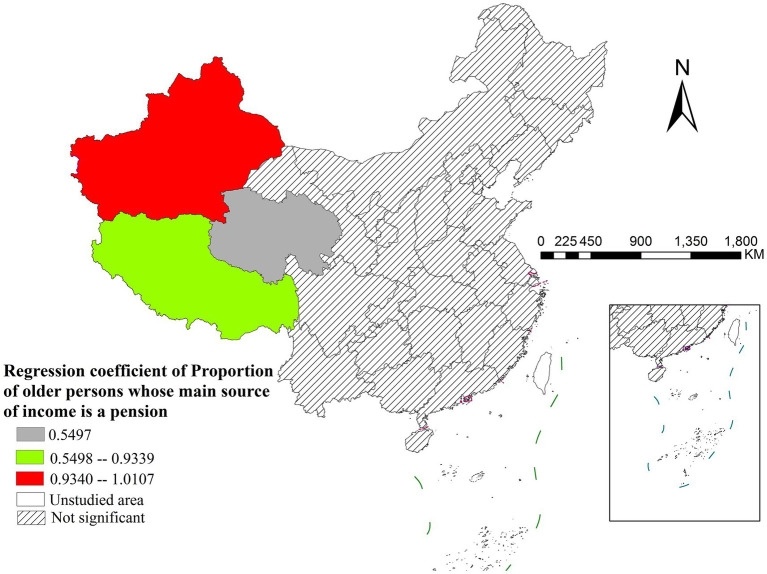
Spatial distribution of regression coefficients for the proportion of older persons whose main source of income is a pension.

**Figure 11:**
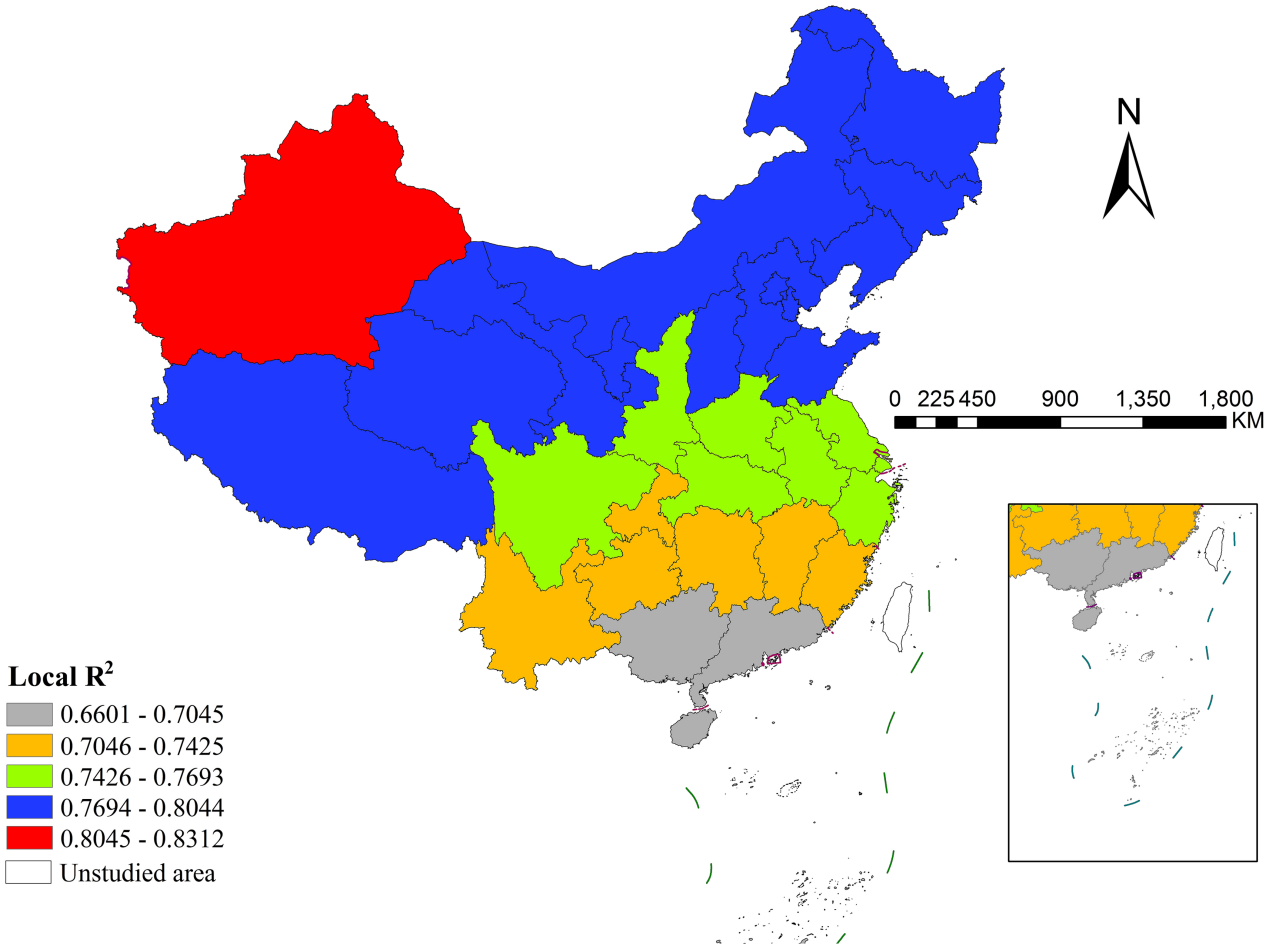
Spatial distribution of local *R*^2^.

Despite the lack of significant spatial dependence detected by global spatial models, local models suggested potential spatial heterogeneity. When comparing the two local spatial regression models, GWR consistently outperformed MGWR across all three goodness-of-fit metrics: GWR achieved a higher *R*^2^, a lower AIC, and a higher Log Likelihood (Table 8). While MGWR is theoretically expected to outperform GWR by allowing variable-specific bandwidths, its iterative back-fitting algorithm may become unstable with small sample sizes (N = 31). The substantially lower Log Likelihood and higher AIC of MGWR suggest potential overfitting or biased bandwidth estimation due to the limited number of provincial units. Therefore, GWR was selected for further interpretation (Figures 7–11). Due to the limited sample size, the GWR findings should be interpreted as exploratory spatial patterns that require independent validation with higher-resolution data (e.g., city or county level).

The regression coefficients for the old dependency ratio were positive across all provinces, indicating a consistently positive association with the outcome variable. A clear north–south gradient was observed, with stronger effects in northern China and weaker effects in southern and southwestern regions (Figure 7).

The number of physicians per 1,000 population also showed positive coefficients, with a spatial pattern similar to that of the old dependency ratio. The effects were more pronounced in northern China and relatively weaker in southern and southwestern areas (Figure 8).

Significant negative coefficients were found for PM_10_ concentration. The detrimental effects were stronger in southern China, particularly in the southwest, and weaker in the northeast and eastern Inner Mongolia (Figure 9).

The proportion of older persons whose main source of income is a pension was significant only in certain local areas. The largest effects were observed in Xinjiang, followed by Tibet and Qinghai, while no significant effects were detected in most other provinces (Figure 10).

The local *R*^2^ values varied considerably across regions. The explanatory power of the selected variables was higher in most northwestern and northern regions but relatively lower in southern and southwestern regions, suggesting that the current model captured the underlying mechanisms better in northern China (Figure 11).

Additionally, the effect of average years of education among the older adults on life expectancy was not statistically significant in any province.

## 4 Discussion

Based on data from the 2015 National 1% Population Sample Survey, this study employed a Bayesian random effects model to estimate age-specific mortality rates and life expectancy among the older adults across 31 provinces in China. The performance of multiple spatial regression models in explaining the social environmental determinants of life expectancy was compared, and the spatial heterogeneity in the influence intensity of these determinants was revealed.

First, regarding the mortality estimation results, the Bayesian random effects model effectively handled the volatility caused by small sample data, leading to more robust mortality estimates (9). Model 2, which incorporated the age-region interaction effect, demonstrated significantly better goodness-of-fit than Model 1, indicating that the patterns of age-specific mortality across provinces were not entirely consistent and that the age effect varied substantially across regions. This finding highlights that the interaction between age and region cannot be ignored when modeling mortality at the provincial scale (39). Sensitivity analysis further confirmed the robustness of the model to prior specifications, thereby enhancing the credibility of the study’s conclusions (27).

Second, the spatial distribution of the regional effect was highly consistent with that of life expectancy among the older adults. Economically developed eastern provinces (e.g., Shanghai and Zhejiang) exhibited lower mortality risk and higher life expectancy, whereas underdeveloped central and western provinces (e.g., Henan, Shanxi, and Tibet) showed higher mortality risk and lower life expectancy, presenting a gradient pattern characterized by “high in the east and low in the west, high in coastal areas and low in inland regions (40).” The three northeastern provinces outperformed the central and western regions, which may be attributed to their higher urbanization levels and relatively well-established healthcare systems. This finding suggests that spatial dependence among regions should be fully considered when formulating future health policies for the older adults.

In the analysis of influencing factors, the global regression models showed that the old dependency ratio was consistently positive and significant across all models, indicating that regions with a higher old dependency ratio tended to have higher life expectancy among the older adults. The positive coefficient for the old dependency ratio could be interpreted in two ways. One plausible mechanism, as noted above, is that regions with a higher old dependency ratio may have more comprehensive older adults care systems and stronger family support networks (41). However, we acknowledge an alternative and more parsimonious explanation: the positive association may simply reflect that longer life expectancy itself increases the proportion of the older adults in the population - i.e., regions with higher older adults life expectancy naturally have a higher old dependency ratio, rather than the dependency ratio being causally related to longevity. This reverse causality is a common challenge in cross-sectional studies and should be considered when interpreting the results. Future longitudinal studies are needed to further explore the direction of this relationship. The positive effect of the number of physicians per 1,000 population was significant in both SEM and SLM, suggesting a beneficial role of healthcare resources in extending older adults life expectancy (42). PM_10_ concentration, average years of education among the older adults, and the proportion of older persons relying on pensions did not show significant effects in the global models, possibly because the effects of these factors were masked by spatial heterogeneity.

The comparison of local spatial regression models showed that GWR exhibited better goodness-of-fit, a lower AIC value and higher Log Likelihood than MGWR in this study. Intuitively, as an extension of GWR, MGWR allows different variables to have different bandwidths and would generally be expected to achieve superior fitting performance (43). However, the superior performance of GWR in this study may be attributed to the following reasons. First, the sample size of this study was 31 provincial-level administrative units, which is relatively small, and the iterative back-fitting process of MGWR with multiple bandwidths may have led to overfitting or unstable estimation under such a small sample. Second, at the provincial scale, the spatial processes of the influencing factors may not differ sufficiently to require multiple bandwidths. Third, the cross-validation bandwidth selection procedure in MGWR involves more parameters, which may introduce bias in bandwidth estimation with a small sample size. In summary, GWR may be a more appropriate choice for small-sample studies at the provincial scale (44).

From the spatial distribution of GWR coefficients, the effects of each variable showed regional variation that warrants further investigation.

The regression coefficients for the old dependency ratio displayed a “high in the north, low in the south” pattern, with a stronger positive association in northern China. One possible explanation, though speculative, is that this pattern could be associated with the closer family structures and higher community-based older adults care coverage in northern regions, which is broadly consistent with the view that northern areas might be able to leverage the advantages of family and community support for older adults health, whereas southern regions might need to pay greater attention to the health risks of older adults individuals living alone or in empty-nest households (45, 46). The effect of the number of physicians per 1,000 population also showed larger coefficients in northern China. A tentative explanation is that healthcare resources are relatively sparse in the north, which could correspond to higher marginal health benefits from an increased physician supply. If confirmed by future studies, less developed northern provinces might consider continuing to strengthen the allocation of primary care physicians, while developed southern provinces could shift from quantity expansion to quality improvement of healthcare services (47). PM_10_ concentration showed negative coefficients, with the largest magnitude observed in southwestern China. This may reflect, as a hypothesis, the complex terrain and poor pollutant dispersion conditions in the region. Based on these exploratory findings, southwestern areas might consider prioritizing air pollution control and establishing health warning systems targeting the older adults population (48). The proportion of older persons relying on pensions as their main source of income reached conventional significance levels only in northwestern China, suggesting as a hypothesis that stable pension income may be associated with older adults health in economically less developed regions. If replicated, northwestern areas could consider continuing to expand pension coverage as a fundamental safeguard for promoting older adults health (49).

The spatial distribution of local goodness-of-fit indicated that the selected variables explained life expectancy among the older adults substantially better in northern China than in southern China, suggesting that additional important influencing factors not included in the model, such as dietary habits, climatic conditions, and social capital, may play a greater role in southern regions.

Several limitations of this study should be acknowledged. First, due to data availability constraints, some potentially important variables such as individual-level lifestyle factors, socioeconomic status, and clinical measures could not be included in the analysis. Second, the cross-sectional design precludes causal inference; therefore, the associations identified in this study should be interpreted as correlational rather than causal. Third, official national statistics are only available at the provincial level, which limits our analysis to 31 provincial-level units; future studies with finer spatial scales such as city or county level are needed to validate the findings. A key limitation is that with only 31 provincial-level units, the spatial patterns observed by GWR may be partly attributable to statistical noise. Our within-sample *R*^2^ improvement does not validate the spatial heterogeneity; independent replication is necessary. Fourth, although the 2015 data avoided the confounding effects of the COVID-19 pandemic, their timeliness is limited, and more recent data should be used in future research to confirm the persistence of the observed spatial patterns. Fifth, death underreporting may vary substantially across regions, with potentially higher rates in western and rural areas due to less developed vital registration systems; however, due to data limitations, we were unable to further adjust for provincial level differences in underreporting. Future research should address this issue by incorporating more accurate death registration data or applying correction methods specifically designed for subnational areas.

The main innovations of this study are twofold. First, methodologically, the integration of the Bayesian random effects model with multi-scale spatial regression approaches not only addressed the robustness of small-sample mortality estimation but also revealed the spatial heterogeneity of influencing factors. Second, in terms of content, the study focused on the older adults population rather than the general population, filling a gap in the existing literature, which has predominantly focused on life expectancy at birth. The findings provide a scientific basis for formulating differentiated older adults health policies and optimizing the allocation of healthcare resources across provinces.

## 5 Conclusion

By integrating the Bayesian random effects model with spatial regression approaches, this study systematically assessed provincial disparities in life expectancy among the older adults in China and their social environmental determinants. The results revealed a pronounced “high in the east, low in the west” spatial gradient in life expectancy among the older adults, highlighting prominent interregional health inequalities. In exploratory GWR analyses, the old dependency ratio and healthcare resource allocation showed positive coefficients that were larger in northern China. Air pollution coefficients were more negative in southwestern China, whereas pension security reached significance only in less developed northwestern regions. These findings underscore the complexity of the mechanisms underlying interregional differences in older adults health and suggest that a uniform national policy may be insufficient to address regional health inequalities. However, given the exploratory nature of the GWR analysis, these spatial patterns require independent validation. If confirmed, each province might consider region-specific strategies for older adults health interventions.

## Data Availability

The raw data supporting the conclusions of this article will be made available by the authors, without undue reservation.
